# The complete plastid genome sequence of *Dracaena fragrans* (L.) Ker Gawl. (Asparagaceae)

**DOI:** 10.1080/23802359.2020.1860719

**Published:** 2021-03-01

**Authors:** Huan Jiang, Yong Kang, Xiaoli Chen, Xiuwei Yang, Xinquan Yang

**Affiliations:** Hainan Provincial Key Laboratory of Resources Conservation and Development of Southern Medicine, Hainan Branch of the Institute of Medicinal Plant Development, Chinese Academy of Medical Sciences and Peking Union Medical College, Haikou, China

**Keywords:** Antimicrobial, Asparagaceae, *Dracaena*, ornamental plant, phylogeny

## Abstract

*Dracaena fragrans* is a shrub native to tropical Africa classified in the family Asparagaceae. It is a common horticultural plant grown as a hedge as well as an ornamental house plant for its green leaves, attractive shape and fragrant flowers. The species has been used for many medicinal purposes including inducing labor, treating malnutrition and fighting illness. Here, we investigated the complete plastid genome of *D. fragrans* using Illumina 150 bp next-generation sequencing technologies. *De novo* assembly and annotation showed that the chloroplast genome is a typical quadripartite structure with 37.5% GC content. A pair of inverted repeats (IRs, 26,507 bp each) were separated by one large single-copy region (LSC, 83,703 bp) and one small single-copy region (SSC, 18,466 bp). The chloroplast genome is 155,183 bp in length and contained 131 genes of which 12 are intron-containing genes, comprising 85 protein-coding, 38 tRNA, and 8 rRNA genes. Phylogenetic analysis of *D. fragrans* and 11 plastomes obtained from GenBank shows that it is closely related to *D. elliptica*.

*Dracaena fragrans* (L.) Ker Gawl., a cornstalk plant that belongs to the family Asparagaceae, is native to tropical Africa and has also been introduced all over the world (Dhar 2013; Banerjee et al. 2017). In Africa, the species is commonly grown as a hedge to help remove indoor pollutants,such as bisphenol A, formaldehyde, toluene, and xylene (Wolverton 1997; Saiyood et al. 2010; Banerjee et al. 2017). It is also popular as ornamental plant used for both indoor decoration and outdoor landscaping in Thailand (Dhar 2013; Banerjee et al. 2017; Julsrigival et al. 2020). As one of the world’s economic plants (Wiersema and León 2013), it has also been used for many medicinal purposes, including inducing labor by squeezing or chewing its leaves and roots (Kamatenesi-Mugisha and Oryem-Origa 2007), treating malnutrition by using a decoction from its bark (Lacroix et al. 2011), and increasing CD4 counts to treat HIV/AIDS by boiling the roots in water (Moshi et al. 2012). In addition to exhibiting significant antimicrobial activity (Lacroix et al. 2011; Banerjee et al. 2017), *D. fragrans* is hypothesized to be an acetylcholinesterase inhibitor (Calderón et al. 2010). Recently, linalool, among the other 29 compounds, was identified in this plant (Julsrigival et al. 2020), revealing its aromatherapy effects for treating inflammation (Peana et al. 2002), inducing relaxation (Linck et al. 2009), sedation (Linck et al. 2010) and relieving anxiety (Guzmán-Gutiérrez et al. 2015). Here, we performed Illumina genome sequencing on a specimen of *D. fragrans* to determine its plastid structure and phylogenetic relationship to other species classified to the genus *Dracaena*.

The specimen of *D. fragrans* was collected from Xinglong Tropical Medicinal Botanical Garden in Hainan Province (18.7345047 N, 110.1940079 E). The voucher specimen (Huan Jiang, JH046) was deposited in the Traditional Chinese Medicine Herbarium of Hainan Province. Total genomic DNA was extracted from fresh leaves dried immediately by silica gel using a modified CTAB method (Doyle and Doyle 1987). Genome skimming was performed by using next-generation sequencing technologies on the Illumina Novaseq 6000 platform and 150 bp paired-end reads with an insert size around 350 bp by Novogene Bioinformatics Technology Co. Ltd. (Tianjin, China). In total, 4.36 Gb of clean data were obtained for assembling the complete chloroplast genome. The GetOrganelle pipeline (Jin et al. 2020) was employed as the *de novo* genome assembly tool and Bandage (Wick et al. 2015) was used to construct the circular assembly. The mean sequencing coverage of this chloroplast genome was 120.5X. The program Plastid Genome Annotator (Qu et al. 2019) was used to annotate all genes with *D. droca* (NC_048492) designated as the reference. The preliminary annotation result was imported into Geneious Prime 2020.1.2 (Biomatters Ltd., Auckland, New Zealand) for manual adjustment of the start/stop codons, intron boundaries and tRNA genes. The plastome was deposited in GenBank under accession number MW123093. To reveal the phylogenetic position of *D. fragrans* within the genus *Dracaena*, 11 complete plastid genome sequences were downloaded from the GenBank, of which 3 species in the genus *Liriope* were used as outgroups. All sequences were aligned using the MAFFT v7.450 algorithm (Katoh and Standley 2013), implemented as a plugin in Geneious Prime with default parameters except for setting the automatic determination of the direction of sequence. A new sequence matrix was generated by multiple alignment to construct the phylogenetic tree. The maximum-likelihood (ML) analysis was performed in RAxML-HPC2 on XSEDE v8.2.10 (Stamatakis 2014) through the CIPRES Science Gateway web server (https://www.phylo.org/) with 1000 Bootstrap iterations under the GTRGAMMA substitution model. The program FigTree v1.4.4 was used to visualize and annotate the phylogenetic tree.

After *de novo* assembly, annotation, and statistical analysis of nucleotide sequences and genes, the plastome of *D. fragrans* displays a typical circular DNA molecule with a total length of 155,183 bp and contains 131 genes (85 protein-coding genes, 8 rRNAs and 38 tRNAs) of which 12 are intron-containing genes (*rps*16, *atp*F, *rpo*C1, *ycf*3, *rps*12, *clp*P, *pet*B, *pet*D, *rpl*16, *rpl*2, *ndh*A, *ndh*B). The chloroplast genome also showed a typical quadripartite structure with 37.5% GC content: one large single-copy region (LSC, 83,703 bp) and one small single-copy region (SSC, 18,466 bp) were separated by a pair of inverted repeats (IRs, 26,507 bp each). Each of inverted repeats contained 7 protein-coding genes (*rps*19, *rpl*2, *rpl*23, *ycf*2, *ndh*B, *rps*7, *rps*12).

The phylogenetic analysis found that species classified in the genus *Dracaena* were divided into two major clades (namely Clade I and Clade II). The *D. fragrans* chloroplast genome sequenced in this study was nested in the Clade I (Figure 1) on a fully supported branch with *D. cochinchinensis* (NC_039943). These two species were practically indistinguishable at the level of chloroplast genome sequence polymorphism, with the exception of only 5 SNPs. However, another sequence (MN200195) of *D. cochinchinensis* was nested into Clade II where species show branch internodes that are typically much shorter than wide, leaves crowded at branch apices, and a base completely covering internode (Chen and Turland 2000). Naturally, *D. cochinchinensis* shares the same characters with the Clade II. Based on this similarity and morphological characteristics, the *D. cochinchinensis* (NC_039943) sequence deposited in the GenBank is likely misidentified. The phylogenetic tree showed *D. fragrans* and *D. cochinchinensis* (NC_039943) are sister to *D. elliptica* (MN200196) in Clade I. Analogously, *D. cochinchinensis* (MN200195) and *D. cambodiana* (NC_039776, MN200194) have the same relationship with *D. draco* (NC_048492) in Clade II. Similar phylogenetic relationships in *Dracaena* were also reported using complete plastid genome sequences (Zhang et al. 2019). The chloroplast genome of *D. fragrans* newly generated here is useful in identifying *Dracaena* species and provide essential data to investigate the systematics of the genus *Dracaena* and related taxa in the Asparagaceae.

**Figure 1.**
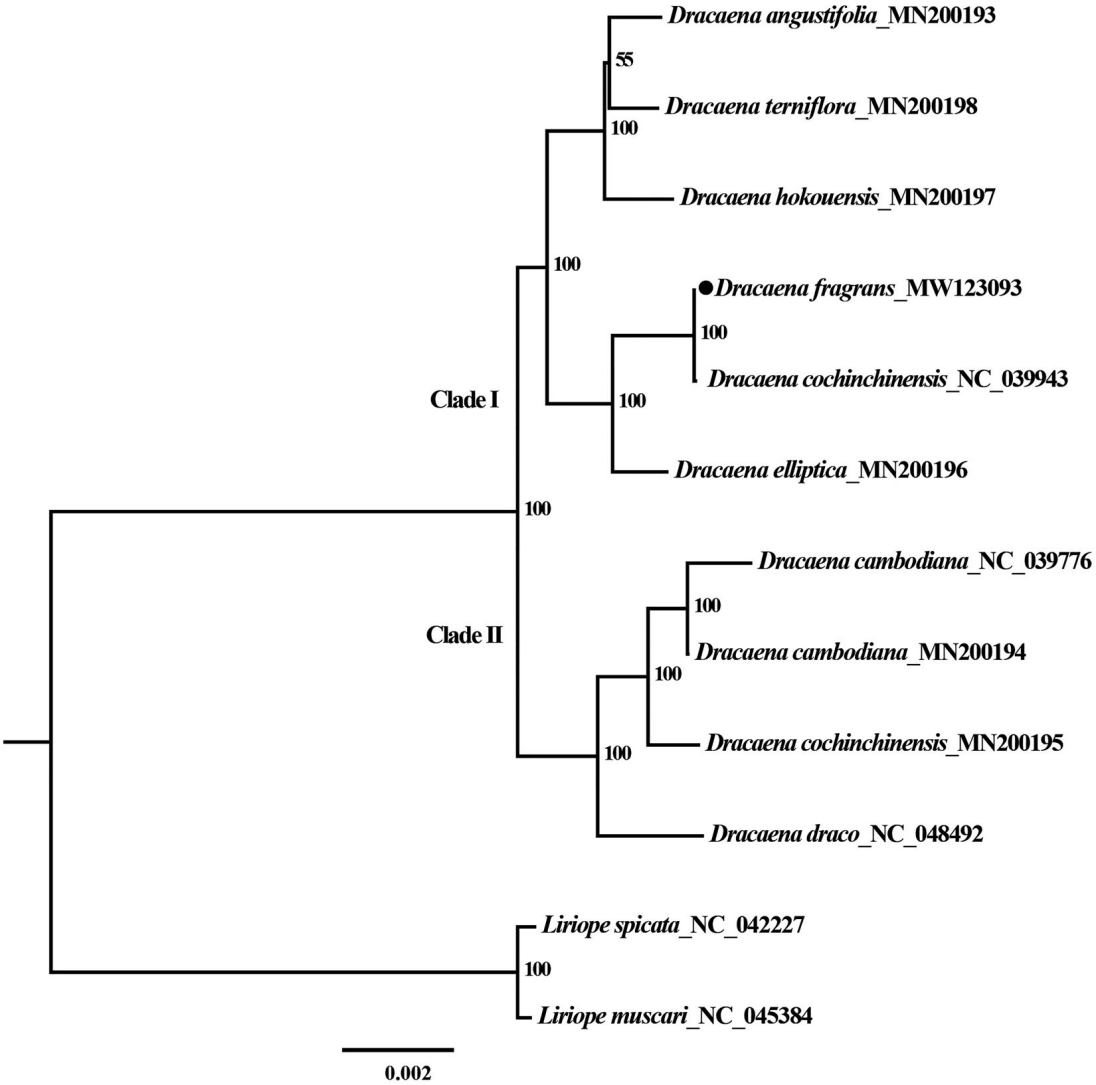
The maximum-likelihood (ML) phylogenetic tree based on the complete plastid genome sequences. Numbers at the right of nodes are bootstrap support values.

## Data Availability

The genome sequence data that support the findings of this study are openly available in GenBank of NCBI at (https://www.ncbi.nlm.nih.gov/) under the accession number MW123093. The associated BioProject, SRA, and Bio-Sample numbers are PRJNA673642, SRR12959444, and SAMN16619521 respectively.
